# Supplementary material for: Observation of Rabi dynamics with a short-wavelength free-electron laser
Source: Nature. 2022 Aug 17;608(7923):488–93. doi: 10.1038/s41586-022-04948-y (PMC9385478; doi:10.1038/s41586-022-04948-y)
Supplement: Supplementary file 1 — This file contains the Supplementary discussion, notes and references. [file 41586_2022_4948_MOESM1_ESM.pdf]

nature portfolio

https://doi.org/10.1038/s41586-022-04948-y

Supplementary information

Observation of Rabi dynamics with a short-wavelength free-electron laser

In the format provided by the
authors and unedited

Supplementary Discussion

Single-atom model

The Rabi amplitudes for two atomic states: $|a\rangle$ and $|b\rangle$, coupled by an oscillating interaction, are given by [1, 2] (atomic units are used unless otherwise stated):

$$a(t) = [\cos(Wt/2) - i(\Delta\omega/W)\sin(Wt/2)]\exp(i\Delta\omega t/2), \tag{S1}$$

$$b(t) = -i\exp(-i\Delta\omega t/2)\frac{\Omega}{W}\sin\left(\frac{Wt}{2}\right) \tag{S2}$$

where the generalized Rabi frequency is $W = \sqrt{\Omega^2 + \Delta\omega^2}$, with Rabi frequency, Ω, and detuning, $\Delta\omega = \omega - \omega_{ba}$, with respect to the atomic resonance energy, $\omega_{ba} = \epsilon_b - \epsilon_a$. The Rabi wave packet: $|\Psi_{\mathcal{R}}(t)\rangle = a(t)\exp(-i\epsilon_a t)|a\rangle + b(t)\exp(-i\epsilon_b t)|b\rangle$ is a solution in the two-level subspace $\mathcal{R}$, spanned by $|a\rangle$ and $|b\rangle$, within the Rotating Wave Approximation (RWA) with the boundary condition $a(0) = 1$ and $b(0) = 0$. Since the RWA is an excellent approximation for the experimental parameters considered [2], $\Omega/\omega_{ba} \approx 0.3\%$, we use it as a zeroth-order solution to our XUV-FEL experiment. In order to obtain corrections, we need to consider the complement to the two-level subspace, which we label: $\mathcal{S}$. These corrections are computed by time-dependent perturbation theory using Dyson equations with coupling to the continuum: $|\epsilon\rangle$ that is part of the $\mathcal{S}$ space and an eigenstate of the atomic Hamiltonian $H_0|\epsilon\rangle = \epsilon|\epsilon\rangle$. In general the exact propagator: U, of the total time-dependent Hamiltonian: $H(t) = H_0 + V(t)$, is not known analytically, but using the Rabi amplitudes in Eqs. (S1)-(S2), we can construct an excellent approximation to the exact propagator in the $\mathcal{R}$ space as:

$$U_{\mathcal{R}}(t,0)|a\rangle = a(t)\exp[-i\epsilon_a(t)]|a\rangle + b(t)\exp[-i\epsilon_b(t)]|b\rangle. \tag{S3}$$

The full propagator can be written as a Dyson equation:

$$U(t,0) = U_{\mathcal{R}}(t,0) - i\int_0^t dt' U(t,t')V_{\perp\mathcal{R}}(t')U_{\mathcal{R}}(t',0) \tag{S4}$$

where the interaction has the internal $\mathcal{R}$-space interaction removed: $V_{\perp\mathcal{R}}(t) = V - RV(t)R$, where R is a projector on $\mathcal{R}$. The field-free atomic propagator:

$$U_0(t', t) = \sum_c U_c(t', t) = \sum_c |c\rangle\langle c| \exp[-i\epsilon_c(t' - t)], \tag{S5}$$

does not couple $\mathcal{R}$ and $\mathcal{S}$ because it is diagonal in the basis of atomic eigenstates: $H_0|c\rangle = \epsilon_c|c\rangle$. The amplitude for one-photon ionization to the continuum can be approximated to lowest order in $V_{\perp\mathcal{R}}$:

$$\alpha_\epsilon(t) = -i\int_0^t dt' \langle\epsilon|U(0,t')V_{\perp\mathcal{R}}(t')U_{\mathcal{R}}(t',0)|a\rangle \approx -i\int_0^t dt'\langle\epsilon|U_\epsilon(0,t')V_{\perp\mathcal{R}}(t')U_b(t',0)|b\rangle b(t'), \tag{S6}$$

where $V_{\perp\mathcal{R}}$ couples from $\mathcal{R}$ to $\mathcal{S}$ within RWA as:

$$\langle\epsilon|V_{\perp\mathcal{R}}(t)|b\rangle = V_{\epsilon b}(t) = z_{\epsilon b}E(t)\exp[-i\omega t]/2. \tag{S7}$$

This result is valid when two photons are required for photoionization: $\omega \approx \omega_{ba}$ and $2\omega_{ba} > I_p > \omega_{ba}$, where I_p is the binding energy of the atom. In this case, the one-photon ionization process from $\mathcal{R}$ to $\mathcal{S}$ is mediated exclusively from the excited state: $|b\rangle$. The final state, $|\epsilon\rangle$, is propagated with U_0, without further interaction with the field in $\mathcal{S}$, as shown on the right-hand side of Eq. (S6). This first-order amplitude can be evaluated analytically as

$$\begin{aligned}\alpha_\epsilon^{(1)}(t) &= -\frac{z_{\epsilon b}E_0\Omega}{2W}\int_0^t dt' \exp[i(\delta_\epsilon - 3/2\delta\omega)t'] \sin\left(\frac{Wt'}{2}\right) \\ &= i\frac{z_{\epsilon b}E_0\Omega}{2W}\left\{\frac{\sin\left[\frac{t}{2}\left(\frac{W}{2} - \frac{3}{2}\Delta\omega + \delta_\epsilon\right)\right]\exp\left[i\frac{t}{2}\left(\frac{W}{2} - \frac{3}{2}\Delta\omega + \delta_\epsilon\right)\right]}{\frac{W}{2} - \frac{3}{2}\Delta\omega + \delta_\epsilon}\right. \\ &\quad \left. - \frac{\sin\left[\frac{t}{2}\left(\frac{W}{2} + \frac{3}{2}\Delta\omega - \delta_\epsilon\right)\right]\exp\left[-i\frac{t}{2}\left(\frac{W}{2} + \frac{3}{2}\Delta\omega - \delta_\epsilon\right)\right]}{\frac{W}{2} + \frac{3}{2}\Delta\omega - \delta_\epsilon}\right\},\end{aligned} \tag{S8}$$

where the relative continuum energy is defined as: $\delta_\epsilon = \epsilon - \omega_{ba} - \epsilon_b = \epsilon - 2\omega_{ba} - \epsilon_a$ with the photoelectron signal being located at roughly: $\epsilon \approx 2\omega + \epsilon_a$, corresponding to $\epsilon^{\text{kin}} = 2\omega - I_p$.

While the lowest-order expression in Eq. (S6) is valid for sufficiently weak XUV-FEL pulses, the present experiment is performed in an intermediate regime where also the second-order interaction with $V_{\perp\mathcal{R}}$ must be investigated. The amplitude for two-photon ionization from state $|a\rangle$, via any accessible intermediate state: $|c\rangle \neq |b\rangle$, can be found in a similar way from the left-hand side of Eq. (S6):

$$\alpha_\epsilon^{(2)}(t) = (-i)^2 \int_0^t dt'' \int_0^{t''} dt' \langle \epsilon | U_\epsilon(0,t'') V_{\perp\mathcal{R}}(t'') U_c(t'',t') V_{\perp\mathcal{R}}(t') U_a(t',0) |a\rangle a(t'), \quad \text{(S9)}$$

where the final and intermediate atomic states are treated to first-order in $V_{\perp R}$ within RWA inside $\mathcal{S}$. We consider only the final energy region close to the two photon excitation energy: $\epsilon \approx 2\omega + \epsilon_a$. The second-order contribution in $V_{\perp\mathcal{R}}$ due to two-photon interaction from the excited state: $|b\rangle$, is neglected since it leads to photoelectrons at higher energies: $\epsilon \approx 3\omega + \epsilon_a$. The amplitude $\alpha_\epsilon^{(2)}(t)$ can be written explicitly as,

$$\alpha_\epsilon^{(2)}(t) = (-i)^2 \frac{z_{\epsilon c} z_{ca} E_0^2}{8} \int_0^t dt'' \int_0^{t''} dt' \exp\left[-i(\epsilon_c + \omega - \epsilon)t''\right] \exp\left[-i(\epsilon_a + \omega - \Delta\omega/2 - \epsilon_c)t'\right]$$
$$\times \left[\exp[iWt'/2](1 - \Delta\omega/W) + \exp[-iWt'/2](1 + \Delta\omega/W)\right]. \quad \text{(S10)}$$

Performing the double integral results in

$$\alpha_\epsilon^{(2)}(t) = -i\frac{z_{\epsilon c} z_{ca} E_0^2}{4} \Bigg\{ \frac{(1 - \Delta\omega/W)\exp\left[i(\frac{W}{2} + \delta_\epsilon - \frac{3}{2}\Delta\omega)\frac{t}{2}\right] \sin\left[(\frac{W}{2} + \delta_\epsilon - \frac{3}{2}\Delta\omega)\frac{t}{2}\right]}{\tilde{\omega}^-(W/2 + \delta_\epsilon - 3\Delta\omega/2)}$$
$$+ \frac{(1 + \Delta\omega/W)\exp\left[-i(\frac{W}{2} - \delta_\epsilon + \frac{3}{2}\Delta\omega)\frac{t}{2}\right] \sin\left[(\frac{W}{2} - \delta_\epsilon + \frac{3}{2}\Delta\omega)\frac{t}{2}\right]}{\tilde{\omega}^+(W/2 - \delta_\epsilon + 3\Delta\omega/2)}$$
$$- \frac{(1 - \Delta\omega/W)\tilde{\omega}^+ + (1 + \Delta\omega/W)\tilde{\omega}^-}{\tilde{\omega}^-\tilde{\omega}^+(\delta_\epsilon + \epsilon_b - \epsilon_c - \Delta\omega)} \exp\left[i(\delta_\epsilon + \epsilon_b - \epsilon_c - \Delta\omega)\frac{t}{2}\right]$$
$$\times \sin\left[(\delta_\epsilon + \epsilon_b - \epsilon_c - \Delta\omega)\frac{t}{2}\right] \Bigg\}, \quad \text{(S11)}$$

where the quantities $\tilde{\omega}^\pm = \epsilon_a + \omega - \epsilon_c - \Delta\omega/2 \pm W/2$ have been introduced. Modulo prefactors, the amplitude $\alpha_\epsilon^{(2)}(t)$ is similar to $\alpha_\epsilon^{(1)}(t)$ in the sense that both are peaked at $\delta_\epsilon = 3\Delta\omega/2 \pm W/2$. We interpret these two peaks as an Autler-Townes doublet, but note that the peaks have different

strengths in general with non-zero detuning. Unlike the first-order AT doublet in Eq. (S8), the second-order AT doublet in Eq. (S11) has the same sign on both peaks. This implies that asymmetric quantum interference effects should be expected when photoelectrons are ejected to the same final state with comparable amplitudes in both processes. In addition, $\alpha_\epsilon^{(2)}(t)$ has a peak at $\delta_\epsilon = \epsilon_c - \epsilon_b + \Delta\omega$ that depends on the intermediate state energy, ϵ_c. The closest state to $1s4p$ is $1s5p$ at 0.3 eV.

Construction of perturbed wavefunction

In order to evaluate the second-order amplitude a summation and integration over all accessible intermediate states should be performed. In this process it is plausible that the ϵ_c-dependent additional peaks of $\alpha_\epsilon^{(2)}$ will wash out due to their different energy positions and phases. The summation and integration over all accessible intermediate states implies a perturbed wavefunction (or "giant wave" from the main text) of the form:

$$|\rho^{\pm}_{\neq b}\rangle = \sum_{c\neq b}\!\!\!\!\!\!\!\int \frac{|c\rangle z_{ca}}{(\epsilon_a + \omega - \epsilon_c - \Delta\omega/2 \pm W/2)}, \tag{S12}$$

which can be identified as part of the first and second term in Eq. (S11). Compared to treating the ionization step using the strong field approximation [3], the method presented here includes the effect of intermediate bound states, but does not include the interaction with the XUV-FEL to all orders for states in $\mathcal{S}$. Thus, the model does not include AC Stark shifts of the two-level system due to coupling to the complement states. Indeed, perturbation theory estimates that they are significantly smaller than the blue shift required to see the symmetric AT doublet in Fig. 3d and Fig. 4d in the main text.

For the model results that are presented in the main article, the perturbed wavefunction was constructed using configuration interaction singles (CIS) basis states for He. When computing the perturbed wavefunction, $\Delta\omega$ and W were neglected in the denominator of Eq. (S12), as they were found to not have a significant impact on the resulting photoelectron spectra. We denote

the perturbed state constructed with these approximations as $|\rho_{\neq b}\rangle$. As shown in Fig. 3(a) of the main article, the radial part of the perturbed wavefunction is significantly larger in magnitude when compared to the radial wavefunction of state $|b\rangle$. The reason for the perturbed wavefunction to be so large is that all intermediate states are similar to each other close to the Coulomb potential. This leads to constructive interference and to a large dipole transition element from the intermediate states to the final state as compared to the transition from the $|b\rangle = 1s4p$ state to the final state. Only two intermediate states ($1s2p$ and $1s3p$) add destructively to the perturbed wavefunction since they have less energy than the $1s4p$ state (as shown in Fig. 3(a) of the main text).

To compute the dipole transition elements to the continuum, CIS continuum functions were used, and the estimated values for a photoelectron kinetic energy of 23.3076 eV can be found in Extended Data Table 1. Combining the factor from one extra interaction with the electric field, $E_0/2$, with the ratio of the dipole elements it is possible to estimate the ratio of the amplitudes for the one- and two-photon processes

$$R^\ell = \left| \frac{E_0}{2} \frac{z^\ell_{\rho\neq b}}{z^\ell_b} \right|, \tag{S13}$$

where z^ℓ_i is the transition matrix element from state i to a final continuum state with orbital angular momentum ℓ. At an intensity of 2×10^{13} W/cm^2, which corresponds to an electric field amplitude: $E_0 = \omega A_0 = 0.023880\,\text{a.u.}$, we estimate $R^s = 0.13546$ and $R^d = 1.1958$. While the one-photon transition is clearly dominant to the s-wave, the two contributions to the d-wave are more comparable in magnitude. The total signal mainly comes from the two-photon transition to the d-wave, but interference with the one-photon transition to the d-wave can not be neglected since it will lead to a blue shift of the symmetric AT doublet. In the main article, the photoelectron spectra shown for the model contain the combined signal for both s and d final states. All atomic parameters required for the model are taken from the ab initio TDCIS

calculations described in Methods.

Effective ionization rate

From the ionization amplitudes it is possible to derive an effective ionization rate that takes into account the contributions from the one- and two-photon processes. For simplicity, we assume resonant photon energy, and that the pulse is long enough to form a well-separated Autler-Townes doublet in the spectrum. Following the procedure that gives rise to Fermi's golden rule [4] leads to an ionization rate for each partial wave ℓ that is the sum of the rates of ionization from $|a\rangle$ and $|b\rangle$,

$$\Gamma_{a+b}^{\ell} = \Gamma_{b}^{\ell} + \Gamma_{a}^{\ell} = \pi \left(\left| \frac{z_b^{\ell} E_0}{2} \right|^2 + \left| \frac{z_{\rho \neq b}^{\ell} E_0^2}{4} \right|^2 \right), \quad \text{(S14)}$$

where the individual rate from each state is reduced by a factor $1/2$ compared to a straightforward application of the golden rule. This factor $1/2$ is understandable, since at resonance each of the two states is populated only half of the time, and is consistent with what has previously been reported for the one-photon process [5]. From the effective rate, we get an estimate of the lifetime as $\tau_{a+b} = 1/\Gamma_{a+b}$, where $\Gamma_{a+b} = \sum_{\ell} \Gamma_{a+b}^{\ell}$ is the sum of the rates for all partial waves for photoionization from both $|a\rangle$ and $|b\rangle$. The lifetime is plotted together with the individual contributions from $|a\rangle$ and $|b\rangle$, denoted τ_a and τ_b, in Fig. 1c of the main manuscript. From TDCIS simulations, we find that the ionization yield in the experiment is within 0.1%. This is consistent with the lifetime estimate from the model (~ 100 ps), which gives an ionization yield close to 0.05% depending on the exact pulse length.

Supplementary Notes

Intensity averaging over macroscopic interaction volume

To better understand the experimental results, we perform an intensity averaging procedure of the analytical model results described above. We assume a Gaussian beam profile with intensity

varying as

$$I(\rho, z) = I_0 \frac{w_0^2}{w(z)^2} \exp\left[-\frac{2\rho^2}{w(z)^2}\right], \tag{S15}$$

with $\rho^2 = x^2 + y^2$, w_0 the beam waist at focus, and $w(z) = w_0\sqrt{1 + (z/z_R)^2}$, where z_R is the Rayleigh length of the beam. We estimate that $w_0 \approx 10.2\ \mu$m and $z_R \approx 6.3$ mm (see, Methods for details). Since the target gas is spread out over a finite volume, the experimental signal will contain the response of atoms subject to a range of different intensities according to Eq. S15. The total signal strength is given by

$$S(\epsilon) = \int_{V_{\mathrm{gas}}} dV |c(\epsilon, I)|^2, \tag{S16}$$

where $c(\epsilon, I)$ is the spectral amplitude for energy ϵ calculated with the model at intensity I, and V_{gas} represents the extent of the gas target. Since the beam waist is much smaller than the extent of the target in the transverse directions, we treat the target as a box shape with an extent of $L = 2$ mm along the z-axis. In the transverse direction we consider contributions that are closer than $5w_0$ from the axis of the beam. The total signal is then

$$S(\epsilon) = 2\pi \int_{-L/2}^{+L/2} dz \int_0^{5w_0} d\rho\rho |c(\epsilon, I)|^2. \tag{S17}$$

The integrals are evaluated using numerical routines from the SciPy library [6].

Extended Data Fig. 6a contains a comparison of the shape of the intensity averaged spectrum and the spectrum for a single atom subject to the peak intensity, at the detuning where the peaks appear symmetric ($\Delta\omega = 62$ meV). Extended Data Fig. 6b and 6c contains the same type of curves for the separate contributions from the two- and one-photon process, but at zero detuning (where they are symmetric), illustrating that the two processes are affected differently by the intensity averaging procedure. The spectra in Extended Data Fig. 6b and 6c are normalized to the maximum of each simulation type (single-atom or macroscopic average). The shape of the two-photon signal is less affected by intensity averaging than the one-photon signal, since

most of the signal comes from areas with high intensity due to the quadratic dependence on the electric field in the amplitude. On the other hand, the one-photon signal should have significant contributions from a larger volume of the target, since the one-photon amplitude scales linearly with the strength of the electric field. This means that the shape of the one-photon signal might become more distorted, as seen in Extended Data Fig. 6c, but that its relative contribution compared to the two-photon process should increase, when compared to the single-atom case.

References

[1] Rabi, I. I. Space Quantization in a Gyrating Magnetic Field. *Phys. Rev.* **51**, 652 (1937).

[2] Autler, S. H. & Townes, C. H. Stark Effect in Rapidly Varying Fields. *Phys. Rev.* **100**, 703 (1955).

[3] Girju, M. G., Hristov, K., Kidun, O. & Bauer, D. Nonperturbative resonant strong field ionization of atomic hydrogen. *J. Phys. B: At. Mol. Opt. Phys.* **40**, 4165 (2007).

[4] Sakurai, J. J. & Napolitano, J. *Modern Quantum Mechanics* (Cambridge University Press, 2017).

[5] Holt, C. R., Raymer, M. G. & Reinhardt, W. P. Time dependences of two-, three-, and four-photon ionization of atomic hydrogen in the ground 1^2S and metastable 2^2S states. *Phys. Rev. A* **27**, 2971 (1983).

[6] Virtanen P. *et al.*, SciPy 1.0: Fundamental Algorithms for Scientific Computing in Python. *Nat. Methods* **17**, 261 (2020).
